# A Rare Case of Lemierre’s Syndrome Caused by Streptococcus pyogenes

**DOI:** 10.7759/cureus.75823

**Published:** 2024-12-16

**Authors:** Mário G Fontoura, Rita P Araujo, Ana G Paupério, Rita Q Costa, Mónica Teixeira

**Affiliations:** 1 Internal Medicine Department, Unidade Local de Saúde Entre Douro e Vouga, Santa Maria da Feira, PRT

**Keywords:** internal jugular vein thrombophlebitis, lemierre syndrome, oropharyngeal infection, septic thrombophlebitis, streptococcus pyogenes

## Abstract

Lemierre’s syndrome (LS) is a rare condition characterized by septic thrombophlebitis of the internal jugular vein (IJV). Typically, the primary infection originates in the oropharynx, progressing to the lateral pharyngeal space, IJV, and potentially leading to bacteremia. Through septic embolization, these patients can develop severe complications, underscoring the importance of early diagnosis.

We present a case of LS in a 33-year-old female who initially presented to the emergency department with odynophagia and was diagnosed with acute tonsillitis. She was discharged with antibiotics. However, one week later, she returned with sudden-onset dyspnea. After a comprehensive workup during her hospital stay, an LS caused by *Streptococcus pyogenes* was diagnosed. The patient was treated with anticoagulation and antibiotics, resulting in a favorable clinical outcome.

This case highlights the importance of maintaining a high level of clinical suspicion for LS in the differential diagnosis of complicated tonsillitis, emphasizing the critical role of early identification and appropriate management. Additionally, we aim to review the pathophysiology, management strategies, and current literature on LS, offering insights into the clinical approach to these rare yet potentially fatal cases.

## Introduction

Lemierre’s syndrome (LS) is a rare condition with an annual incidence of 3.6 cases per million people [1]. First described in 1936 by French professor André Lemierre, it is characterized by septic thrombophlebitis of the internal jugular vein (IJV) [2]. While it predominantly affects young, healthy males, cases in elderly individuals have also been reported [3-5].

In most instances, the primary infection is oropharyngeal, involving the tonsils or peritonsillar tissue, with subsequent local invasion to the pharyngeal space and IJV, resulting in septic thrombophlebitis within one to three weeks. The microbiological agents are often commensals of the oropharyngeal flora, most commonly *Fusobacterium necrophorum*, followed by *Enterobacteriaceae*, *Streptococcus* spp., and *Staphylococcus *spp. [2,5]. The mechanism by which the infection spreads from its initial focus to the intravascular space is not fully understood, though some authors postulate that prior viral infections may contribute to mucosal damage, facilitating bacterial dissemination by direct tissue invasion; other postulated mechanisms are the hematogenous spread and immune modulation [6].

The clinical presentation is nonspecific and includes fever, odynophagia, dysphagia, trismus, and unilateral neck pain, often accompanied by swelling and induration of local muscle masses. Common pulmonary complications include pneumonia, lung abscesses, and pleural empyema [7].

Diagnosis is typically established through imaging studies demonstrating IJV thrombosis and identification of a causative microorganism from blood cultures or pharyngeal exudate cultures. Computed tomography (CT) of the neck and chest is the preferred modality as it also helps rule out pulmonary complications, though neck ultrasound is an alternative [8].

Treatment of LS is primarily based on antibiotic therapy, and delayed initiation is a significant risk factor for mortality. Prompt empirical antibiotic therapy targeting anaerobes is critical and should be adjusted based on microbial sensitivity results [9,10]. According to some studies,* F. necrophorum* shows variable susceptibility to different antibiotics, with the combination of β-lactams with β-lactamase inhibitors or metronidazole and clindamycin in penicillin-allergic patients being the treatment of choice [5,10]. However, no specific guidelines exist regarding the most appropriate antibiotic regimen [5,10,11]. The duration of therapy varies from two to four weeks to up to 20 weeks, depending on disease severity, and should continue until clinical and radiological findings have resolved [5]. Once infection control is achieved, therapy can be completed orally. Treatment may also include surgical intervention, primarily as abscess drainage (indicated for large abscesses >2 cm, abscesses located near vital structures with possible airway compromise, or failure to respond to antibiotics within 48-72 hours) and/or anticoagulation [5].

Regarding anticoagulation, current evidence is controversial. While less severe presentations (without massive thrombotic phenomena) usually do not require anticoagulation, it may be recommended in cases with extensive thrombosis, multi-organ involvement, or progressive clinical deterioration [10]. In such cases, patients with clinical improvement may transition to oral anticoagulation, usually for up to three months [12]. In cases where thrombus resolution is suboptimal or symptoms persist despite antibiotic therapy, a longer period of anticoagulation may be necessary. Frequent assessment of bleeding and thrombosis recurrence risks is critical to determine the optimal treatment duration [13].

Although LS is rare, it is potentially fatal, with mortality rates ranging from 6% to 15% [14]. Early recognition is therefore essential.

This case report explores the critical features of this condition and emphasizes the need for heightened clinical awareness when evaluating acute tonsillopharyngitis with persistent neck pain, particularly regarding its potential complications.

## Case presentation

We present the case of a 33-year-old Caucasian woman, autonomous in activities of daily living, with a relevant medical history of a spontaneous miscarriage at 37 weeks of gestation, for which a comprehensive evaluation for hereditary and acquired thrombophilia was negative. She was on regular medication with dienogest and ethinylestradiol (2 mg + 0.03 mg daily) and reported no drug allergies.

The patient first presented to the emergency department with a two-day history of odynophagia. A diagnosis of viral acute pharyngitis was made, and she was discharged with symptomatic treatment. Initially, her symptoms improved; however, after one week, she developed sudden-onset dyspnea and right hypochondrial chest pain radiating to the ipsilateral dorsolumbar region, which occurred nocturnally while sleeping. She returned to the ED. Epidemiological history revealed no recent exposures, travel, or contact with sick individuals. At this time, she denied experiencing any other symptoms, including neck pain. On physical examination, she was hemodynamically stable but tachycardic and tachypneic at rest, without accessory muscle use. Cardiac and pulmonary auscultation revealed no significant findings and abdominal palpation elicited discomfort in the right hypochondrium without a positive Murphy’s sign. There was no peripheral edema.

Complementary studies revealed sinus rhythm tachycardia on electrocardiogram and no other significant findings. Arterial blood gas analysis on room air showed compensated respiratory alkalosis without hypoxemia or elevated lactates. Bloodwork showed normocytic, normochromic anemia (Hb 12.2 g/dL) without other hematological abnormalities, an elevated INR of 1.2 (normal value: <1.5), markedly elevated D-dimers at 30,230 ng/mL (normal value: <500 ng/mL), a cholestatic liver profile with hyperbilirubinemia (R-factor: 1.2), and elevated inflammatory markers (C-reactive protein: 273 mg/L; procalcitonin: 0.38 ng/mL). Chest X-ray revealed a small right-sided pleural effusion. Abdominal ultrasound showed no abnormalities or free fluid. CT angiography of the thorax, abdomen, and pelvis ruled out pulmonary thromboembolism but described a small right pleural effusion and marginal splenomegaly. SARS-CoV-2 antigen testing was negative, and blood cultures turned positive after nine hours, though the Gram stain was not yet available. Urinary pneumococcal antigen was negative, and serologies for hepatitis A, B, and C; HIV; *cytomegalovirus* (CMV); and *Epstein-Barr* virus (EBV) were pending. An infectious and immune workup was initiated. The patient was admitted to the internal medicine ward for management of bacteremia of unclear etiology and started on empirical ceftriaxone (2 g daily).

On the second day of hospitalization, she remained tachycardic and febrile, with worsening inflammatory markers. Overnight, she developed new-onset hypoxemic respiratory failure and dorsal pain. A transthoracic echocardiogram revealed only a thin pericardial effusion without hemodynamic compromise and no evidence of endocarditis. She was transferred to the Intermediate Care Unit.

**Figure 1:**
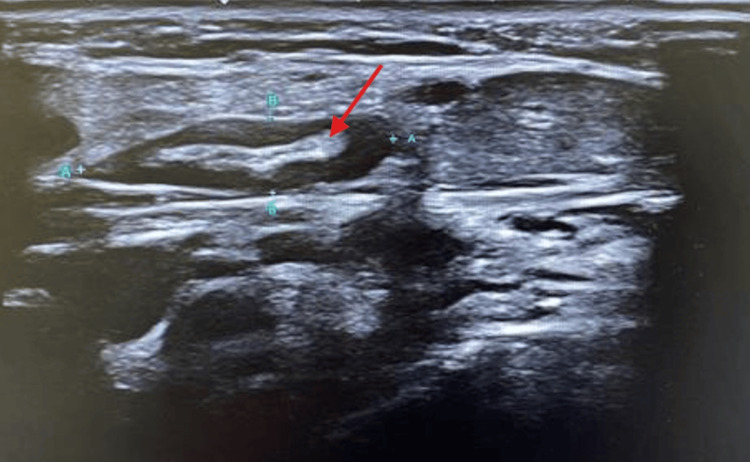
Ultrasound image of the right neck showing an echogenic structure (thrombus, red arrow) within the lumen of the internal jugular vein (delimited by letters A and B, in blue)

On her first day in the unit, she reported new-onset right-sided neck pain. Physical examination revealed a sub-centimeter cervical lymphadenopathy in the anterior chain. By then, blood cultures identified *Streptococcus pyogenes* (group A). Results of the zoonosis panel were negative, and serologies confirmed immunity to hepatitis B, with no evidence of acute hepatitis C, HIV, CMV, or EBV infection. Antinuclear antibodies and immunoglobulins were within reference ranges. Suspecting LS, a soft-tissue neck ultrasound was performed, revealing evidence of recent thrombosis of an internal jugular collateral vein with focal thrombus extension into the IJV without occlusion (Figure 1). A diagnosis of LS was established based on recent tonsillitis, IJV thrombosis, and isolation of a plausible pathogen. Antibiotic therapy was adjusted to benzylpenicillin (400,000 U) based on microbial sensitivities, and therapeutic anticoagulation with enoxaparin (1 mg/kg bid) was initiated. With clinical improvement, she was transferred back to the internal medicine ward.

The patient remained hospitalized for seven days, showing favorable clinical and analytical progression with hemodynamic stability. She was discharged to a Home Hospitalization Unit (HHU), where she completed a 14-day antibiotic course (with follow-up blood cultures showing negative results) and transitioned to edoxaban (60 mg daily) for anticoagulation. She was discharged from the HHU with instructions to continue anticoagulation for three months due to provoked venous thromboembolism and was referred for follow-up in the Internal Medicine outpatient clinic.

To date, the patient remains asymptomatic and free of any complications.

## Discussion

As previously emphasized, early diagnosis of LS is critical for preventing complications and improving outcomes [1]. Despite general consensus regarding the main features of LS, diagnostic criteria are not standardized, which can delay or complicate the diagnosis.

Early diagnosis is challenging as initial symptoms are nonspecific and may mimic those of common oropharyngeal infections. Diagnosis is further complicated in the absence of typical IJV thrombosis signs such as tenderness and edema, as was the case with this patient. Additionally, the rarity of LS may contribute to diagnostic oversight among clinicians. In our patient, *S. pyogenes* (Group A) was identified as the causative pathogen, which posed an additional diagnostic challenge due to its uncommon association with LS, having been documented in only a limited number of cases [15]. Imaging findings of IJV thrombosis solidified the diagnosis, though initial clinical findings could have easily been attributed to a lower respiratory tract infection.

Management of LS requires a multidisciplinary approach. Resuscitation and antibiotic therapy remain the cornerstones of treatment and should be individualized [7]. Empirical therapy should target *F. necrophorum* with β-lactams plus β-lactamase inhibitors or carbapenems, combined with metronidazole in cases of β-lactam allergy [5,10]. In this case, empirical therapy was initially ceftriaxone due to suspected zoonosis. After identifying *S. pyogenes*, therapy was adjusted to penicillin, and a 14-day course was completed. The ideal duration of antibiotic therapy remains debated, with recommendations ranging from two to six weeks [16,17].

Anticoagulation in LS is controversial and utilized in up to 56% of cases [2,18]. While it may prevent thromboembolic complications, its safety profile is uncertain due to potential bleeding risks [5,13]. Recent studies suggest anticoagulation-related bleeding is rare in LS, which does not inherently increase hemorrhagic complications [2]. In this case, anticoagulation with low-molecular-weight heparin and subsequent transition to edoxaban was well-tolerated, with no bleeding events. Current guidelines recommend individualizing anticoagulation decisions based on thrombus location, disease progression, and bleeding risk. The optimal duration of anticoagulation remains unknown. In this patient, a three-month course was chosen based on provoked thrombotic events and close outpatient follow-up.

## Conclusions

This case highlights the need for heightened clinical awareness of LS in patients presenting with persistent oropharyngeal infections and bacteremia of unclear origin, particularly with new-onset neck pain and IJV thrombosis. Early multidisciplinary management of this potentially life-threatening syndrome is essential, with prompt diagnosis and timely treatment being essential to improving outcomes. Future research efforts should prioritize the development of standardized diagnostic criteria and management guidelines for this rare but potentially life-threatening syndrome.
